# Harnessing Stakeholder Perspectives and Experience to Address Nutrition Risk in Community-Dwelling Older Adults

**DOI:** 10.3390/healthcare9040477

**Published:** 2021-04-16

**Authors:** Catherine B. Chan, Naomi Popeski, Leah Gramlich, Marlis Atkins, Carlota Basualdo-Hammond, Janet Stadnyk, Heather Keller

**Affiliations:** 1 Diabetes, Obesity and Nutrition Strategic Clinical Network, Alberta Health Services, Calgary, AB T2W 1S7, Canada; naomi.popeski@ahs.ca; 2 Department of Agricultural, Food and Nutritional Science, University of Alberta, Edmonton, AB T6G 2E1, Canada; 3 Department of Physiology, University of Alberta, Edmonton, AB T6G 2H7, Canada; 4 Department of Community Health Sciences, University of Calgary, Calgary, AB T2N 1N4, Canada; 5 Department of Medicine, Division of Gastroenterology, University of Alberta, Edmonton, AB T6G 2G3, Canada; lg3@ualberta.ca; 6 Nutrition Services, Alberta Health Services, Edmonton, AB T5J 3E4, Canada; marlis.atkins@ahs.ca (M.A.); Carlota.basualdo@ahs.ca (C.B.-H.); janet.stadnyk@ahs.ca (J.S.); 7 Department of Kinesiology, Schlegel-University of Waterloo Research Institute for Aging, University of Waterloo, Waterloo, ON N2G 0E2, Canada; hkeller@uwaterloo.ca

**Keywords:** undernutrition, malnutrition, community-dwelling older adults, community organizations, healthcare delivery

## Abstract

Community-dwelling, older adults have a high prevalence of nutrition risk but strategies to mitigate this risk are not routinely implemented. Our objective was to identify opportunities for the healthcare system and community organizations to combat nutrition risk in this population in the jurisdiction of Alberta, Canada. An intersectoral stakeholder group that included patient representatives was convened to share perspectives and experiences and to identify problems in need of solutions using a design thinking approach. Results: Two main themes emerged from the workshop: (1) lack of awareness and poor communication of the importance of nutrition risk between healthcare providers and from healthcare providers to patients and (2) the necessity to work in partnerships comprised of patients, community organizations, healthcare providers and the health system. Conclusion: Improving awareness, prevention and treatment of malnutrition in community-dwelling older adults requires intersectoral cooperation between patients, healthcare providers and community-based organizations.

## 1. Introduction

A high rate of malnutrition in hospitalized patients is a long recognized clinical issue. Recently the Canadian Malnutrition Task Force (CMTF) reported that 45% of patients who stayed in hospital more than two days were malnourished at admission [1] and most remained in this condition when discharged [2]. Moreover, older adults (≥65 years) represent approximately 40% of the hospital population, thus malnutrition may be disproportionately impacting this population [3]. This high level of malnutrition is also seen on admission to hospital, which suggests that the cause may not be related to hospital care but rather due to a lack of awareness and resources in the community [2].

Using standardized methodology and cut-points, a tri-country study (Canada, New Zealand, The Netherlands) found a uniformly high prevalence of high nutrition risk (61.5–70.1%) in community-dwelling adults ≥65 years of age [4]. The validated screening tool (SCREEN II) is considered useful for identifying upstream factors (e.g., determinants of food intake such as grocery shopping difficulty) that may predict future as well as current malnutrition [5]. The term nutrition risk is used here to denote this upstream view of risk. The United Kingdom Malnutrition Task Force reported that 93% of people at risk of malnutrition or malnourished are living in the community, the remainder being in care homes or hospital [6]. The consequences of this high prevalence of malnutrition are increased rates of hospitalization and admission to residential care as well as an overall reduction in quality of life [7,8]. It is estimated that approximately 30% of seniors who enter residential care could have remained at home with appropriate community supports [9]. Because there are typically substantial numbers of people in acute care settings awaiting placement to residential care, it is important to find innovative ways to reduce this burden on the health care system.

Factors that contribute to malnutrition in community-dwelling seniors include psychosocial and environmental or economic contributors such as lack of transportation, poverty, or living alone [10,11,12]. Additionally, physiological factors such as chronic health conditions, oral health and medication use [12] may be contributing factors. As their health declines and individuals receive homecare services, indicators such as frequency and intensity of pain, or assistance with toileting may also predict nutrition risk [13]. While earlier recognition of nutrition risk is desirable, screening in primary care settings is underutilized [14]. Reluctance to implement screening may include clinical barriers such as how to identify target populations (e.g., focusing on pre-frail or frail patients) to maximize benefit, lack of knowledge of where the at-risk patient can be referred, or patient barriers such as unwillingness to follow through with a referral [15].

Several current initiatives in Alberta, Canada align with mitigating nutrition risk in community-dwelling older adults. The Diabetes, Obesity and Nutrition (DON) Strategic Clinical Network™ (SCN™) identified malnutrition in this population as a strategic priority in its 2015 Transformational Roadmap [16]. The Primary Healthcare Integration Network™ (PHCIN™) focused on hospital to home transitions in its strategic priorities [17]. Nationally, the CMTF developed guidance documents, including an adaptable clinical care pathway to optimize nutrition (care) for older adults living independently in the community [18]. However, specific, contextually-tailored interventions need to be proposed for each region, due to differences in services available.

**Figure 1:**
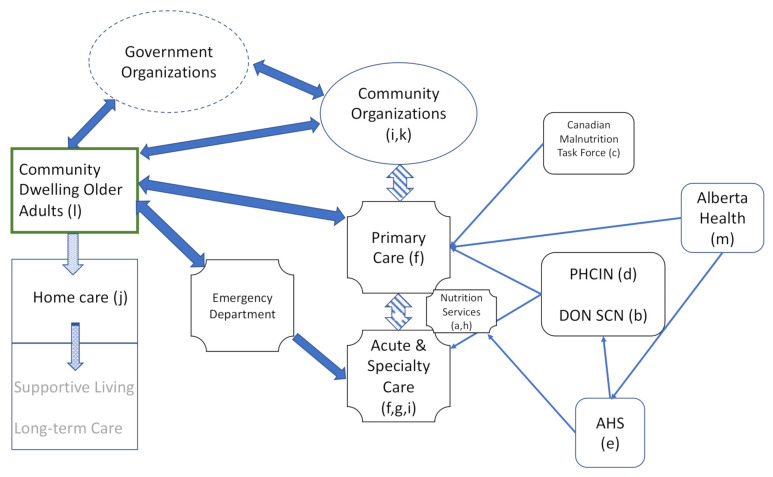
Constituencies of knowledge users and their connections to community-dwelling older adults (green box), the healthcare system and other organizations. Solid dark blue arrows denote direct interaction of older adults with the healthcare system, government and community organizations. Stippled arrows show potential progression of older adults to requirement for home care support or long-term care. Thick striped arrows indicate the relationship between acute and primary care with community organizations, which was identified by symposium participants. It was also identified that these connections could be strengthened, for example through transitions pathways from acute to primary care. Community organizations identified that many of their services could be better utilized by augmenting connectivity between themselves and primary care. Thin arrows indicate organizations that provide policy and other leadership support for implementation of best practices; Alberta Health provides operating budgets to the primary care system and AHS. Involvement of other government organizations not directly providing healthcare was not explored in depth. Letters in parentheses reference Table 1.

Canada has a publicly funded healthcare system providing hospital, specialist, emergency and primary care as well as certain ancillary services [19]. In the province of Alberta, Canada, Alberta Health Services (AHS) is the single health system with responsibility for acute and continuing care, as well as Nutrition Services, relevant to the current study. Nutrition Services provides provincial leadership in development and implementation of policy and practices for all nutrition-related activities, from population and public health to acute and primary care (see Figure 1). Community organizations also provide health-related services that help older adults maintain independent living, such as provision of meals or transportation. Funding models for such programs may include user-pay or subsidies through government or non-government grants. Although pilot projects have been implemented, in which patients discharged to home are “prescribed” meal delivery with costs covered by a grant [20], in general food services are not publicly funded.

Spearheaded by the DON SCN™ and taking an integrated knowledge translation (iKT) approach, these organizations plus other key stakeholders came together to (1) share current information about and create empathy for older adults experiencing nutrition risk and malnutrition, particularly in Alberta; (2) brainstorm solutions; and (3) develop a framework for intervention development using a design thinking process. Methodologies for iKT are not systematically chosen, implemented or evaluated but meetings and presentations are commonly employed [21]. AHS’ Design Lab has a mandate to catalyze organizational change by having stakeholders co-create solutions using design thinking (www.d4ahs.com, accessed on 23 March 2021). The purpose of design thinking is to help stakeholders solve difficult problems, with the first step being creating empathy around the problem by engaging with the population in need, reviewing current literature, and considering the socioeconomic context and other factors when developing potential solutions [22]. The focus of this iKT event was to facilitate brainstorming and developing solutions for malnutrition in older adults that align with the knowledge users’ knowledge and experiences; however, the diverse range of stakeholders was first apprised of the current state from multiple expert viewpoints in order to break down siloed thinking and create empathy. This report highlights how the iKT process resulted in identification of new directions for preventing and treating nutrition risk and malnutrition in community-dwelling older adults.

## 2. Materials and Methods

The Research Ethics Board at the University of Alberta approved this study entitled “Malnutrition Symposium and Workshop” (Pro00097622). Prior to attending, participants were provided with an information letter indicating that the symposium and workshop were part of a research project. The information letter was reviewed at the beginning of the symposium and participants were given the opportunity to opt out by communicating with the organizers at any time up to one month following the event. The research took place in three phases following a design thinking protocol.

### 2.1. Overall Structure of the Integrated Knowledge Translation Activities

Integrated knowledge translation, as defined by the Canadian Institutes of Health Research, is “an approach to doing research that applies the principles of knowledge translation to the entire research process” [23]. By involving stakeholders in research as collaborators, it was anticipated that the research outcomes would be more applicable to knowledge users. Thus, a symposium (Day 1) and workshop (Day 2) was convened to involve stakeholders in a design thinking process to articulate why the problem of malnutrition in older adults exists as well as potential solutions. The stakeholders included patients, healthcare professionals and administrative leadership, community organization representatives and researchers. This methodology was preferred over other techniques that were considered, such as modified Delphi processes using questionnaires as was used by CMTF to develop an in-hospital nutrition pathway [24], because the face-to-face format offered better opportunity for building new connections between people and organizations.

### 2.2. Participants

Targeted invitations were sent to more than 70 individuals (Table 1). Invitations were sent by email approximately two months prior to the Symposium and Workshop, with additional follow-up reminders. If invitees were unavailable, they could suggest another knowledge user from their organization. Registration was free but limited by the available budget; therefore, efforts were made to ensure attendance from the various constituents. On arrival at the venue, participants signed in to log attendance and were given their group assignment for Phase 2 activities described in Section 2.3.2. Patient representatives were provided an honorarium in recognition of their contribution. Attendance from geographic regions was encouraged by providing transportation and hotel accommodation.

### 2.3. Design Thinking

AHS supports innovation via several embedded processes, including learning collaboratives and design thinking. Design thinking was selected as a framework for tackling the problem of malnutrition in older adults because of its focus on the people impacted by the condition and their needs [25] and it allowed for multiple approaches to solve problems, whereas the learning collaborative model is focused specifically on pathway-driven quality improvement. This problem-based research approach meets criteria for iKT, which is an approach that engages knowledge users at multiple phases of the research and implementation of potential solutions. Participating in co-design with knowledge users in the research process can help disseminate findings that are applicable and meaningful because of the unique insights brought to the findings. The process also facilitates the movement of research results into health practices or programs and/or policy [26]. The overall structure of the Malnutrition Symposium and Workshop was developed with input from AHS’ Design Lab personnel.

#### 2.3.1. Phase 1—Evaluating Current State (Empathize)

In order to understand the current state of caring for older adults with malnutrition in Alberta, on Day 1 the symposium organizing committee enlisted presenters from a variety of constituencies as shown in Table 2. Podium presentations were delivered to cover background information about malnutrition in older adults in Canada, the expertise and capacity present in community organization, the activities of the CMTF and the transitions in care pathway work of the PHCIN™. Those on the panel discussion provided brief explanations of the role of their constituency in responding to nutrition risk in older adults, followed by a question-and-answer session. Panel member presentations and discussions were audio-recorded and transcribed verbatim. During a world café formatted session, presenters spoke briefly on ongoing initiatives with small groups of participants. Attendees also had the opportunity to learn more about PHCIN™ and CMTF work on transition pathways. These discussions were recorded on paper by note-takers.

#### 2.3.2. Phase 2—Brainstorming Solutions (Define the Problem, Ideate)

In groups of 6–8 representing the different constituencies and individuals not usually working together, symposium attendees were charged with developing two problem statements regarding malnutrition in older adults in Alberta, building on their own experience in addition to the information provided in Phase 1 and alignment with the research priorities of the day. For each problem statement, the groups then brainstormed and prioritized co-created potential solutions. Discussions were recorded on paper by note-takers. At the end of Day 1, the symposium organizing committee and the Design Lab facilitators used the notes to discuss impressions of the day and to develop six problem statements to use for the Workshop in Phase 3.

#### 2.3.3. Phase 3—Developing a Framework for Intervention Development (Ideate, Prototype)

The activities on Day 2 were facilitated by Alberta Health Services Design Lab personnel (https://www.d4ahs.com, accessed on 23 March 2021) according to their protocol. In small groups, workshop participants selected a problem statement and, over six hours, developed an intervention that addressed the stated problem. These interventions form the framework for future development and refinement of interventions for older adult nutrition risk and malnutrition.

### 2.4. Analysis

Recordings from Phase 1 presentations, discussions and question and answer periods were transcribed verbatim and organized in Excel as strengths and gaps in nutrition care for older adults. Themes were developed inductively by iterative categorization of the concepts and ideas presented by the speakers, during the world café and question periods as recommended for thematic analysis following the steps of familiarization, coding, generating initial themes, reviewing themes, defining/naming themes [27]. This was carried out by C.B.C. and checked for accuracy and consistency by N.P. Any disagreements were resolved by discussion and consensus. Quotations were sorted by themes, e.g., (Potential) Role of Government/AHS, Primary Healthcare, Community Organizations; User/Patient Perspective; Relationship of Malnutrition to Socioeconomic Determinants of Health; Strategies. Reports from Phase 2 brainstorming were also transcribed and synthesized. The transcripts were examined for mention of linkages and strategies used by different constituencies that already existed and/or could be strengthened to enable continuity of care for older adults with nutrition risk, such as better transitions and community support.

## 3. Results

### 3.1. Participants

Attendance for the symposium and workshop is reported in Table 1.

### 3.2. Current State

The purpose of Phase 1 was to provide an understanding of the problem of nutrition risk in community-dwelling older adults in Alberta, so that participants realized the scope of the problem, developed empathy for those at risk and were focused on identifying solutions. Speakers in Phase 1 were charged with describing the current state of identifying and caring for seniors in Alberta who are malnourished or at nutrition risk, and perspectives on gaps in the system that needed to be addressed. Presenters were representatives of several constituencies in order to capture multiple points of view (Table 2 and Figure 1). Speakers identified a number of existing initiatives; however, these were largely occurring independently without formalized cooperation or partnership between constituents. Overall, a key message emerged related to lack of knowledge and communication of malnutrition, not only to older adults but also to healthcare professionals who are not dietitians. Secondly, malnutrition and nutrition risk are closely related to social isolation and other social determinants of health as well as clinically relevant conditions such as frailty. Scrutiny of the transcripts identified strengths and gaps in addressing nutrition risk in older adults—summarized in Table 3.

**Figure 2:**
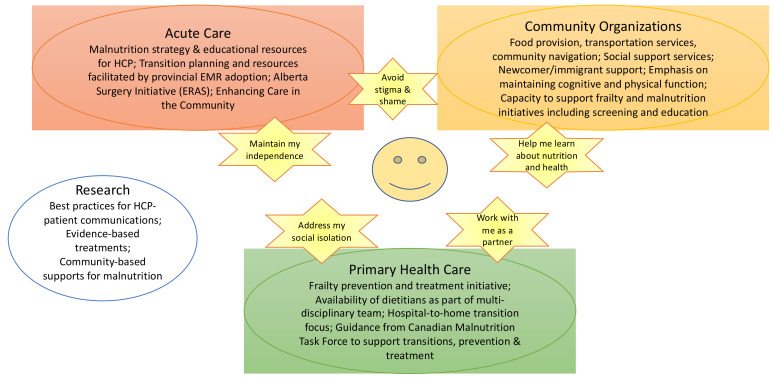
Identification of initiatives that could be leveraged to address nutrition risk in community-dwelling older adults. These ideas were derived from the transcripts of the Symposium’s presentations and discussions. The figure depicts a patient-centred approach to anchor initiatives and programs. A need for intersectoral cooperation was identified, for example in facilitating transitions from hospital (acute care) to home (primary health care and community organizations) as was the existence of already present supports such as the AHS Malnutrition strategy and the CMTF pathways for transitions and primary care. Research areas to support evidence-based practice were identified. Abbreviations: EMR, electronic medical record; HCP, healthcare professionals.

In addition to identifying general strengths and gaps in the current state, additional presentations focused on initiatives already underway in AHS, primary care settings, academic and community organizations (Figure 2). Within Alberta, ongoing efforts to support the malnutrition strategy and recent roll-out of a provincial electronic medical record that will facilitate charting of malnutrition and its identification as a discharge planning and care transition issue were identified by Nutrition Services as critical to advancing continuity of care for patients with malnutrition risk. In addition, initiatives outside of Nutrition Services that could be enhanced further by “adding on” nutritional assessment and treatment were identified. These included the PHCIN™ guidance on home to hospital to home transitions, CMTF-led development of a pathway for addressing screening and support for patients with nutrition risk in the community, a focus on frailty prevention by the Senior’s Health Strategic Clinical Network™. Community organizations identified a number of ongoing food provision initiatives as well as related services such as transportation and mitigating loneliness. Community organizations emphasized that while they have some capacity to assist with the problem of malnutrition, they find themselves competing for limited resources rather than presenting a unified, coordinated approach. Discussion focused on how these activities could be leveraged to enhance nutrition-related care for older adults.

### 3.3. Prioritizing the Most Important Gaps, Solutions, Barriers and Facilitators to Solutions

Following the presentations outlined in Table 2 along with ample opportunity for question-and-answer and general discussion, participants were organized into six groups from diverse constituencies for Phase 2—Brainstorming Solutions. Each group was charged with identifying two of the most important gaps or problems based on their own knowledge and the information provided during the presentations. The key problems arising were:

- Lack of awareness and importance of (mal)nutrition among older adults. This problem was related to physical function (“…patients don’t connect nutrition with function … and so therefore it’s not really a priority in maintaining their health”).
- Ineffective communication of malnutrition between HCP-HCP and between HCP and patients. This was exemplified during open discussion by this quote, “I have a hard time making a diagnosis about malnutrition or telling the patient that they have malnutrition…. maybe we have to change our language but I can’t believe that we cannot help patients… like if you had cancer you would say ‘Oh my God that’s going to make them feel bad. I better not tell them that.’” One of the groups stated, “Then our last problem statement is: there’s no standardized assessment or communication or treatment plans across health care providers. And so, we talked about having the importance of kind of common … language and more research to continue on the patient’s perspective and how they view this…. We all felt that would…help bring these things forward.”
- Lack of health system recognition of and education in the importance of nutrition and nutrition risk in older adults (“…care providers don’t view malnutrition as some of those asymptomatic problems that they need to address or act on.”)
- Diagnosing physical changes with aging (chewing, swallowing, dentition) that affect nutrition as exemplified by this quote: “We talked a lot about the chewing and swallowing issues, people don’t want to admit that they have them and that they’re doing fine. They think needing modified texture is a failure. And that those people are often readmitted for malnutrition and aspiration; so again, just catching those readmissions and identifying that and working with the patients.”
- Lack of assessment of nutrition risk and continuity of care in the health system (“…there’s no standardized assessment or communication or treatment plans across health care providers.”)
- Social isolation among older adults as a driver of nutrition risk, represented by the quote: “…people who are lonely are more likely to be malnourished…”

Once the problems had been articulated, the same groups were asked to propose solutions. The most common solution was education and awareness campaigns aimed at older adults and their families, volunteers and organizations who work with older adults, the general public and HCP to address the gap in knowledge of nutrition, malnutrition and its link to maintaining function and independence in older adults. The importance of ensuring that older adults participate in the development of such a campaign was emphasized. A second solution was aimed at addressing the communication gap, in particular reconciling the stigma of malnutrition might confer patients versus their need to understand the importance of proper nutrition to maintaining their health and independence. A third solution was ensuring that malnutrition was assessed for, diagnosed and charted for the purposes of discharge planning, transitions and continuity of care, and appropriate treatment. Finally, the important roles that community organizations already play in safeguarding the health of older adults was acknowledged and ideas advanced for extending those roles and for creating better connections between government, health, community and academic sectors.

The last activity of Phase 2 was identification of problem statements that would be used in Phase 3—Developing a Framework for Intervention Development. This task was facilitated by the AHS Design Lab team. The symposium and workshop organizing committee (i.e., the authors) reviewed the gaps and problems and selected six, which the facilitators further refined to address the patient-centered approach that would be taken in Phase 3.

### 3.4. Developing a Framework for Interventions

On Day 2, the participants were organized into groups representing multiple constituencies and led through a process of identifying a problem from Day 1 for which they would develop a prototype solution. The facilitators had purposely worded the problem statements to emphasize the centrality of the patient with nutrition risk. The AHS Design Lab facilitators challenged participants to develop a prototype intervention within a 6-h window of time via interactive small group activities and large group discussions. The interventions and the problems they address are listed in Table 4. Notably all of the six solutions involved partnerships of the health system with community-based organizations and some also included increased connectivity between acute and primary care as well as other government departments. Although there were only three patient representatives present, they had input into all of the interventions through a process in which members of each group learned about the innovation of each other group and offered feedback and suggestions for strengthening the proposed solution.

## 4. Discussion

Approximately two-thirds of older adults in Canada are identified as having nutrition risk [4]. It is recognized that better screening as well as identifying opportunities for prevention could help older adults retain their independence and improve their quality of life as well reduce hospital length of stay and associated costs. Leveraging the initiatives of several stakeholders, an iKT event was undertaken using a design thinking process to increase empathy for older adults with nutrition risk and malnutrition amongst multiple stakeholders, brainstorm solutions and develop ideas for future intervention development.

A high-functioning health system puts patients and patient-centered care at the center of all activities, including innovation. Our Symposium and Workshop included three patient representatives, one of whom was a speaker on the panel discussion. In addition, these individuals are part of AHS’ Patient and Community Engagement Researcher (PaCER) team, and have been trained to conduct qualitative, patient-oriented research to support innovation in healthcare. They had previously conducted a project with the DON SCN™ to interview people recently discharged from hospital with a diagnosis of malnutrition. Thus, they had an excellent perspective as exemplified by the panel discussion presenter (Table 2). The patient representatives brought three important themes from their research, patient unawareness of malnutrition risk, barriers to eating well as well as personal facilitators such as trying new recipes, consulting dietitians, sharing meals with friends. Our findings are consistent with the priorities identified in a citizen panel comprised of older adults and specifically the need to improve malnutrition through accessing knowledge about food and nutrition, identification of nutrition risk in older adults and coordinated services delivery [28]. The United States National Blueprint: Achieving Quality Malnutrition Care for Older Adults, 2020 Update also targets educating older adults and their caregivers on nutrition-related topics and services [29].

Lack of awareness of the importance of good nutrition in maintaining physical and cognitive function and independence, not only by patients but also by the healthcare system was identified as a key problem. Health system priorities such as optimizing hospital-to-home transitions, a provincial surgery initiative and implementing care pathways for nutrition risk in primary care settings were recognized as opportunities to leverage initiatives such as enhanced screening for and follow-up of nutrition risk in community settings. Conversely, community organizations identified opportunities for partnership to capitalize on initiatives and services they were already providing to their constituents. Thus, partnerships between community organizations and the health system became a focus of the Workshop on Day 2, when prototype solutions were developed.

The importance of nutrition to overall health and function in older adults was a fundamental knowledge gap identified by multiple stakeholders. Moreover, communicating malnutrition from both conceptual and diagnostic perspectives was determined to be problematic. The proposed communication solution between HCP and patients was a smartphone app that the HCP could program with a specific plan to improve nutrition risk and other health behaviors. The app could also link to community resources and was envisaged as an aid to transitioning from hospital to home. While many currently available apps track nutrition and fitness, mainly to aid weight loss, few seem designed to provide holistic support. An app called WittyFit combining behavioral data with health outcomes is currently in clinical trials [30]; such apps may become more available in future and may be feasible to use in older adult populations as they gain more familiarity and expertise with smartphone technology, but their current use is limited by low uptake by older adults [31].

Capacity within the nongovernmental, community organization sector was identified as a strength on Day 1, as was the opportunity to leverage ongoing initiatives in primary healthcare, AHS-led partnership called Enhancing Care in the Community, the CMTF, and hospital-based programs such as Enhanced Recovery After Surgery [32]. Creation or formalization of intersectoral partnerships could address persistent gaps in nutrition care for older adults because the health concerns of the rapidly growing older adult population in Canada and elsewhere are challenging for traditional healthcare delivery models to provide quality care. Indeed, the World Health Organization’s Active Ageing: A Policy Framework identified the need to “develop a continuum of affordable, accessible, high-quality and age-friendly health and social services that address the needs and rights of people as they age” but the follow-up Report on Ageing and Health noted that little progress had been made over the subsequent decade [33].

Participants addressed how nutrition-related services could be provided in partnership with the healthcare system to provide older adults with more seamless care. Four of the proposed solutions involved enhanced access to food, or reducing social isolation at mealtimes either through group activities or videoconferencing. Community-based supports have been acknowledged as potential strategies for improving nutrition status of older adults living independently [34]. Many of these are informal, involving family, friends and neighbors, while community services providing congregate dining or meal delivery are also recognized to reduce nutrition risk [34]. Two recommended strategies to mitigate nutrition risk in older adults are delivered meals and congregate dining [35]. Post-hospitalization meal delivery has the potential to reduce emergency department visits and re-hospitalization [36]. Congregate dining may concomitantly reduce social isolation and increase nutrient intake [37]. The community organizations present at the Symposium emphasized that many programs already available could specifically target older adults with nutrition risk, including social supports, a variety of meal provision options, transportation and shopping. However, the small volume of published evidence in this area makes it difficult to be guided by best practice. The prototype solutions devised using the Design Lab process used the knowledge gained at the Symposium to suggest practical outreach activities that could be implemented in a variety of settings or use technology to overcome access challenges, particularly in rural areas.

The final solution addressed the problem of lack of knowledge of the importance of nutrition to avoiding conditions such as frailty. Importantly, the older adult voice was considered essential to developing an awareness campaign that would resonate with that audience. We note that none of the solutions focused on communications between healthcare professionals, despite this being an important issue identified at the Symposium. This may have been due to the Design Lab’s focus on patient-centered solutions as well as the interests of individuals attending the Workshop.

The literature is sparse on formalized community-healthcare partnerships. Despite recommendations to improve access [29], models of partnerships still need to be developed. Lim et al. [38] proposed new models not centered around doctors and illness are required; rather, the reforms should focus on integration, patient-centeredness, health-centered, emphasizing team-based care that evolves with the patient from being well-healthy, to well-unhealthy, unwell-unhealthy (frail) and end-of-life care. A specific model developed in Singapore involving the public, businesses, community as well as healthcare organizations was described [38]. Such case studies are extremely useful, but each environment needs to develop strategies and partnership agreements that reflect the local culture and needs. However, an interesting component of the Singapore model was the development of wellness community collectives, which serviced residents by offering programming and courses in physical activity, nutrition, social engagement activities and health related activities—including screening and interventions [38].

With the impetus engendered by this iKT activity, AHS and its partners have started on a path towards intersectoral support for older adult’s nutrition risk or malnourished living in the community. One year post-event, the organizing committee is aware of at least three research projects initiated or proposed that involve academic-health system-community organization partnerships. In addition, plans to adapt and adopt the CMTF-developed pathway for screening for nutrition risk in primary care, with community organization and AHS partnership, are well underway.

## 5. Conclusions

A design thinking protocol was used to identify potential interventions that could be used to create awareness of, prevent or treat malnutrition in community-dwelling older adults. Partnerships between stakeholders, including the voice of patients, were deemed essential and included in this iKT. Our findings indicate that leveraging existing initiatives could reduce barriers to innovation and the new intersectoral partnerships can support a way forward for addressing nutrition risk and malnutrition in older adults in Alberta.

## Figures and Tables

**Table 1:** Constituencies invited and represented at the Symposium and Workshop.

Constituency *	Role	Individuals Invited (*n*)	Individuals Attending Symposium (*n*)	Individuals Attending Workshop (*n*)
Nutrition Services leadership team (a)	Development and implementation of nutrition-related policy and practice	5	5	4
DON SCN™ leadership team (b)	Prioritizing and implementing innovation in healthcare	6	6	5
CMTF (c)	Promoting nutrition care knowledge and optimal practice in prevention, detection and treatment of malnutrition	1	1	0
Other SCNs (PHCIN™ (d), Bone and Joint SCN™, Seniors Health SCN™)	Prioritizing and implementing innovation in healthcare	10	1	1
Academic researchers from provincial universities	Research in malnutrition, frailty, seniors health	9	3	2
AHS administrative leadership (e)	Portfolios such as seniors heath, home care, community liaison	5	2	0
Physicians (f)	Care of community-dwelling older adults	5	2	0
Geriatricians (g)	Specialty care of older adults	4	1	0
Primary/family care network representatives	Provision of comprehensive patient care	4	1	1
Dieticians (h)	Administrative and patient care roles in primary and home care	6	6	6
Allied health professionals (i)	Pharmacists, nurses with focus on care for older adults	3	1	1
Homecare (j)	Administrative and front-line patient care	5	2	2
Community organizations (k)	Provision of services and resources to older adults	6	5	2
Patient representatives (l)	Patient-researchers from the Patient and Community Engagement research team involved in malnutrition research	3	3	3
Indigenous wellness program	Provision of health services to Indigenous clients	2	0	0
Alberta Health, Alberta Housing and Seniors) (m)	Provincial government ministries responsible for policy development and implementation	2	2	2
Supplemental health insurer	Support for seniors health and wellness programs	1	1	1
Municipality	Healthy aging strategy	1	0	0
Trainees	Graduate student, MD resident, dietetic interns	5	5	4

* Many invitees represented more than one constituency but were only counted once. Letters in parentheses refer to constituencies represented in Figure 1.

**Table 2:** Presentations at the Symposium.

Presentation Content	Constituency Represented by the Presenter	Length	Format
Malnutrition in older adults: Prevalence, costs, case study	DON SCN™	15 min	Podium
Keynote theme: “Knowing and not doing is the same as not knowing”; the importance of partnership, sustainability & engagement to address complex problems	Community organization	45 min	Podium
Improving primary care nutrition—developed Integrated Nutrition Pathway for Acute Care; identified better practices for nutrition care in the community; developed Nutrition Care Pathways for Primary Care	CMTF	30 min	Podium
Malnutrition in Alberta: What does it look like? What are we doing? What could we do better? Initiatives mentioned included: assessment of nutrition risk in continuing care clients; patient-centred research and patient perspectives; community drop-in and outreach programs (ways to provide meals, social interaction, roles for churches, community leagues, partnerships with seniors apartment buildings), AHS Nutrition Services malnutrition strategy (raising awareness, prevention, detection and treatment)	Patient representative; AHS Continuing Care; Research; Community organization; AHS Nutrition Services	60 min	Panel discussion
Home to Hospital to Home Transition Guideline—goal is to improve patient quality of care, safety, patient experience, and provider satisfaction. Adaptable to specific care requirements such as nutrition risk	PHCIN™	30 min	Podium
Inspiring initiatives as exemplars: A website of resources directed at older adults (“Seniors Community Hub”) and frailty screening in primary care; AHS-facilitated *Enhancing Care in the Community* initiative to enable Albertans to be as healthy, well and independent as possible in their homes and communities; a seniors organization with a health clinic run by a nurse practitioner	Primary care physicians; AHS community liaison leadership; Community organization; CMTF; PHCIN™	60 min	World Café

Abbreviations: SCN™, Strategic Clinical Network; DON, Diabetes, Obesity and Nutrition Strategic Clinical Network; AHS, Alberta Health Services; CMTF, Canadian Malnutrition Task Force.

**Table 3:** Current state strengths and gaps.

Strengths	Gaps	Quotes
Provincial malnutrition strategy in place with activities focused on acute care and homecare; tools and resources available; information & education available	Implementation of and support for the malnutrition strategy in primary care settings	HCP#1: I was a home care dietitian for about 15 years before I went into leadership, and I have seen these people in their homes struggling to eat well and I don’t know how to fix it necessarily and I think we have to think outside our healthcare walls and really work with partners to understand what is the best approach here.
Provincial hospital-based EMR with capacity to identify and record malnutrition risk	Scaling EMR use to all sites; not applicable to primary care setting; malnutrition not necessarily included in hospital-to-home transition planning	HCP#1: So what we do have [is] the Canadian nutrition screening tool, which is for adults embedded within our new electronic medical record. It’s not across the province yet, but the questions are in Connect Care [EMR] and the same tool is used on paper in other settings across the province.
Nutrition risk assessment of all home care clients	Lack of holistic, long-term palliative care approach for older adults (not focused solely on disease treatment); silos of primary care and acute care	HCP#2: …embedding a palliative approach means looking at not 36 h of palliative care but 36 months of palliative care and how that can be harmonized. …I really worry that her diabetes is overtreated, her hypertension is overtreated, she’s having falls, she’s going to end up with a broken hip and require a nursing home and if there isn’t a general assessment of her whole condition, and part of that is that what we call harmonizing the therapy and the palliative care. …care focused on her quality of life, and her comfort and avoiding acute issues, will enlarge. The therapy, the therapeutic focus part will reduce… we know a single focus on a problem leads to problems. If all you worry about is somebody’s blood pressure, they’ll be on blood pressure pills til the end of their life. If all you worry about is malnutrition, there will be people that want to have people dying of dementia on heart healthy diets.
Community organizations provide programs including food provision, transportation, social engagement	Lack of consensus on best practices in malnutrition/nutrition risk treatment; lack of validated models for partnerships between healthcare system and community organizations	CP: Another program that I’ve used and have been involved in is community dining, or wheels to meals program. And they can be held in churches, community centres, I came from—[name of town] and we used to run them in our neighborhood associations, which are your community leagues (great places to have community dieting programs)… and I have seen a lot of good things come out of that because a lot of the times people that are coming are socially isolated. They’re not eating properly so we get together we eat socially because we’re all social people. We like to eat with people and a lot of the times if people have lost somebody that that they’ve been cooking for or they’re eating partner well then we know what happens and they don’t eat like they used to.
Community organizations have outreach and education capacity and expertise and are connected to other institutions (municipalities, religious organizations, etc.)	Communicating malnutrition diagnosis—what it is, importance—to older adults. Understanding the role of food in maintaining health and speeding recovery from illness	Pt: OK so in this case, this particular person said and this is their perception of malnutrition: “I see a picture of a starving Biafran child. With huge belly, skinny arms, old man face. I am not starving obviously because I am overweight. I am living in [name of town], access to food, knowledge of food, how can I be malnourished? No connection between me and that child.”
Evidence of cost to system of malnutrition on length of stay and readmission to acute care in Canada	Communicating nutrition’s importance in healthy aging	Pt: I also learned the importance of eating and food in health and recovery and think that’s very profound information that I want to disseminate to the world and say, “We have to make food far more important in healthcare and in living than it is right now and I think you’ll find that’s really supported by our data.”
Compared with medical interventions, an individual receiving assistance from a community organization programs can be less stigmatizing	Patient-centred approaches to malnutrition care need to be developed throughout the health system	HCP#3: And we learned that dietitians feel very valid reasons, there are definite reasons why they don’t want to tell patients that they’re malnourished. One, they don’t feel that patients understand what malnutrition means. They are very worried that if they tell a patient that they’re malnourished and it’s stigmatizing, that it’s going to shut down the conversation, instead of open it up for treatment.
Opportunity for leveraging existing initiatives e.g., Enhanced Recovery After Surgery, Hospital-to-Home transitions, primary care as medical home, national malnutrition pathways initiative	Older adults’ lack of knowledge of nutrition; fierce independence; feelings of shame, guilt, stigma	Pt: So then we also … found people feeling shamed and embarrassed by realizing that they were malnourished, but this was their own perception because they hadn’t been told. “So by not taking care of myself the way I used to do it, I’m making things worse. I’m inducing the deficiencies that I know I have. And guilt--pretending to my family that everything is all right. The last thing I need is my family to know is that I am potentially in trouble.”
Primary care via PCNs provides team-based, interdisciplinary care including dietitians, physio- and occupational therapists; includes majority of general practice and family medicine MDs throughout Alberta	Older adults’ lack of knowledge of or access to community supports, particularly in rural settings and by immigrants	CP: Rural Alberta is underserviced, especially when it comes to outreach services and I think that’s a problem because we know that our older adults in rural Alberta do not want to move into the urban. They want to stay in their farmhouse; so how are we going to help mitigate that?
	Use of technology for mass education, program delivery versus older adults’ utilization of such technology	Res: So I think we need to figure out those connection points and what will be feasible and realistic depending on who that senior is, as well as the community provider, too, right, in terms of their technology. I don’t have the answers, but that’s a key part of it is figuring that piece out. And tech’s gotta be at our side; we have to use it better than we have been.

Abbreviations: CP, community partner; EMR, electronic medical record; HCP, healthcare professional; PCN, primary care network; Pt, patient; Res, researcher.

**Table 4:** Problem Statements, Adapted from Problem Statements Generated at the Symposium (Phase 2), Ideas for Solutions Developed During the Workshop (Phase 3), and the Intersectoral Connections Required to Enable the Solutions.

Problem Definition	Prototype Solution	New Connections Proposed
There is a lack of communication and continuity of management of malnutrition between different supports in the community and in the health system; People don’t connect nutrition with phyical and cognitive function	FUNction app—a personalized nutrition, fitness, wellness and sleep plan with information and prompts provided on an app. Supported by a clinician/coach	Acute care-primary care adaptation and uptake of CMTF transition pathway; primary care-community organizations providing services to support the health plan, as well as health coaches
People do not connect nutrition and physical and cognitive function and primary care team members may be unaware of community supports	AWARENESS CAMPAIGN	Co-designed by primary care, community organizations, patients and caregivers
Older adults may be isolated and not have supports to enable them to eat enough or identify that they are malnourished	Meal Makers; Collective Kitchen for Seniors: congregate shopping and cooking at community centres, learning skills and gaining social supports TABLE TALK! Making connections over meal/snack times through videoconferencing	Primary care adapts CMTF pathway (possibly in partnership with community organizations) and recommends appropriate community organizations, programs and resources to patients at nutrition risk
Social determinants of health can have serious impact on senior’s nutrition	STOCK THE BOX! Excess food from grocery stores or community households donated to a central, community-based facility that anyone can access	Primary care, social services, seniors and housing, and community organizations

## Data Availability

Data are available upon reasonable request to the corresponding author.
